# A Hybrid Indoor Altimetry Based on Barometer and UWB

**DOI:** 10.3390/s23094180

**Published:** 2023-04-22

**Authors:** Minghao Si, Yunjia Wang, Ning Zhou, Cheekiat Seow, Harun Siljak

**Affiliations:** 1 Key Laboratory for Land Environment and Disaster Monitoring, China University of Mining and Technology, Xuzhou 221116, China; 2 Chinese Academy of Surveying and Mapping, Beijing 100036, China; 3 School of Computing Science, University of Glasgow, Glasgow G12 8RZ, UK; 4 School of Engineering, Trinity College Dublin, The University of Dublin, D02 PN40 Dublin, Ireland

**Keywords:** indoor positioning, altimetry, barometer, UWB

## Abstract

Accurate altimetry is essential for location-based services in commercial and industrial applications. However, current altimetry methods only provide low-accuracy measurements, particularly in multistorey buildings with irregular structures, such as hollow areas found in various industrial and commercial sites. This paper innovatively proposes a tightly coupled indoor altimetry system that utilizes floor identification to improve height measurement accuracy. The system includes two optimized algorithms that improve floor identification accuracy through activity detection and address the problem of difficult convergence of *z*-axis coordinates due to indoor coplanarity by applying constraints to iterative least squares (ILS). Two experiments were conducted in a teaching building and a laboratory, including an irregular environment with a hollow area. The results show that our proposed method for identifying floors based on activity detection outperforms other methods. In dynamic experiments, our method effectively eliminates repeated transformations during the up- and downstairs process, and in static experiments, it minimizes the impact of barometric drift. Furthermore, our proposed altimetry method based on constrained ILS achieves significantly improved positioning accuracy compared to ILS, 1D-CNN, and WC. Specifically, in the teaching building, our method achieves improvements of 0.84 m, 0.288 m, and 0.248 m, respectively, while in the laboratory, the improvements are 2.607 m, 0.696 m, and 0.625 m.

## 1. Introduction

Indoor localization is a key focus of research in artificial intelligence (AI) and the Internet of Things (IoT) as 80% of people’s daily lives are spent indoors. Several technologies have been developed to achieve accurate indoor localization, including Wi-Fi [1], Bluetooth (BLE), magnetic sensors [2], ultra-wideband (UWB) [3], and visible light communication [4]. While accurate two-dimensional indoor localization is achievable using these technologies, there is currently no robust and environment-independent method for measuring indoor height. Existing studies only provide floor identification or imprecise altimetry, making it essential to develop precise indoor altimetry. This is especially important in irregular structures, such as buildings with hollow areas, commonly found in various industrial and commercial settings. In commercial buildings, precise altimetry is critical for location-based promotions and advertisements, navigation services in train stations and airports, and tracking and navigation of patients and equipment in hospitals [5]. For emergency services such as first aid, rescue, firefighting, and public security, accurate altimetry is necessary, as the Federal Communications Commission (FCC) requires wireless operators to provide vertical (or *Z*-axis) position data. In industrial buildings and facilities, accurate altimetry is crucial for tracking and navigating vehicles, robots, and personnel. In conclusion, precise indoor altimetry is essential in both commercial and industrial buildings.

Existing methods can be divided into two categories based on their ability to provide accurate height: floor identification and altimetry. The first category is mainly based on matching algorithms, such as k-nearest neighbours (KNN), by creating an offline database to match and identify floors in the online phase. Fang Zhao et al. [5] proposed a hybrid floor identification method that extracts the distribution of wireless access points (APs) in different floors to identify the initial floor through Bayesian classification. The floor information obtained from wireless AP distribution is then used to calibrate the barometric pressure-based floor identification to compensate for variable environmental effects. However, the anti-interference ability of Wi-Fi signals is weak, especially in complex indoor environments; particularly in commercial buildings with hollow areas, it is difficult to guarantee their identification accuracy. Some researchers also used vision-based methods to detect floors, as described in [6,7]. However, these methods usually require the creation of a large image database, which is time-consuming and labor-intensive. Moreover, the camera on smartphones is typically monocular, which limits the accuracy of floor identification. Finally, barometers are widely applied in floor identification, but their performance can vary significantly between mobile phones due to equipment heterogeneity. In addition, the accuracy of barometer-based methods is heavily influenced by environmental factors, and parameter adjustment can be time-consuming in different environments [8,9].

The second category is mostly based on barometers or TOF-based sensors. In the indoor positioning competitions of IPIN (indoor positioning and indoor navigation), some teams studied height estimation methods based on barometers [10,11,12,13]. However, these barometer-based methods are often severely affected by barometric drift, and it is difficult to maintain positioning accuracy for a long time. To solve the problem of barometric drift, Bao et al. [14] set up another reference base station to update the reference barometric pressure in real-time. However, most commercial environments currently do not have extra reference base stations, and communication between devices may also limit the widespread adoption of this method. To avoid setting up a new reference base station, Li et al. [15] fused the estimated altitude based on the barometer and the estimated altitude based on UWB using the extended Kalman filter to achieve high-precision altitude measurement. However, the estimated altitude based on UWB is obtained through the triangle center-of-mass algorithm, which sometimes fails to converge when the indoor UWB base stations are coplanar, resulting in inaccurate altitude estimation. In addition, J. Geng et al. [16] used the geometric information of building stairs to estimate height using PDR and a barometer. Two estimated heights were obtained and then combined using the robust adaptive Kalman filter (RAKF) algorithm to calculate the optimal height. However, the barometer and accelerometer sensors in smartphones are known to have low precision, which makes this method unreliable in providing an accurate height. Additionally, some methods rely on laser [17] and inertial measurement units (IMUs) [18]. While both can provide accuracy at the centimeter level, their high cost limits their widespread use.

Overall, the fusion of multiple sensors to achieve high-precision indoor altitude measurement has gradually become a recognized solution by researchers both domestically and abroad. Among these many sensors, barometers have always been a hotspot in the research of traditional indoor altitude measurement, while UWB has also gradually attracted attention due to its high accuracy, high transmission rate, low power consumption, and strong multipath resolution. However, altimetry based on UWB faces limitations caused by indoor anchor-coplanar environments.

In previous height measurement solutions, floor recognition based solely on barometers often required additional reference stations to eliminate the influence of barometric drift. In contrast, in solutions based on the fusion of multiple sensors, accuracy is often improved by inputting the estimated height based on different sensors into a filter. Different from existing research techniques, we have designed a novel indoor altimetry method based on UWB and barometers, in which the floor recognition module can achieve accurate floor recognition over a long period of time without relying on additional sensors. Unlike most fusion solutions, our height measurement system uses the floor recognition results based on a barometer to assist the UWB height measurement instead of mixing estimated heights from different sensors. The contributions can be concluded as follows:

(1) Through analyzing the changes in air pressure during walking horizontally or going upstairs and downstairs, an activity detection module is added to assist floor identification, addressing equipment heterogeneity and barometric drift. This module is designed to be lightweight and can be easily run on commodity smartphones.
(2) After analyzing the observation equation, we identified the mechanism of altimetry error in indoor anchor-coplanar environments based on iterative least squares (ILS). We found that the iterative process often has convergence problems due to specific constraints, which result in errors. To overcome this issue, we improved the altimetry methods based on ILS by carefully selecting an appropriate initial coordinate and implementing iterative control to optimize the convergence process.
(3) Unlike most of the existing methods based on multisensor fusion that mix height data from various sensors, we proposed a scheme that utilizes floor recognition results to assist UWB height measurement. Our method relies solely on barometers for floor recognition, and then improves UWB height measurement based on the floor recognition results using the proposed constrained iterative least-squares method.
(4) To validate the effectiveness of the proposed method, we conducted experiments in both a teaching building and an office building. We compared the proposed activity-detection-based floor identification algorithm (ADFI) and the constrained iterative least-squares-based hybrid altimetry algorithm (CILS-HA) with three other benchmarks, respectively. The results of the experimental campaign demonstrate that our method outperforms the other baseline methods in all scenarios.

This paper is structured as follows. Section 2 clarifies the problems of the barometer-based floor identification method and explains the mechanism of altimetry error based on ILS. Section 3 proposes a novel altimetry method based on barometers and UWB that is suitable for indoor anchor-coplanar environments. In Section 4, the performance of the proposed altimetry method is evaluated. Finally, Section 5 presents the discussion and future work.

## 2. Theoretical Analysis and Problem Descriptions

### 2.1. Barometric Drift and Equipment Heterogeneity

**Figure 1:**
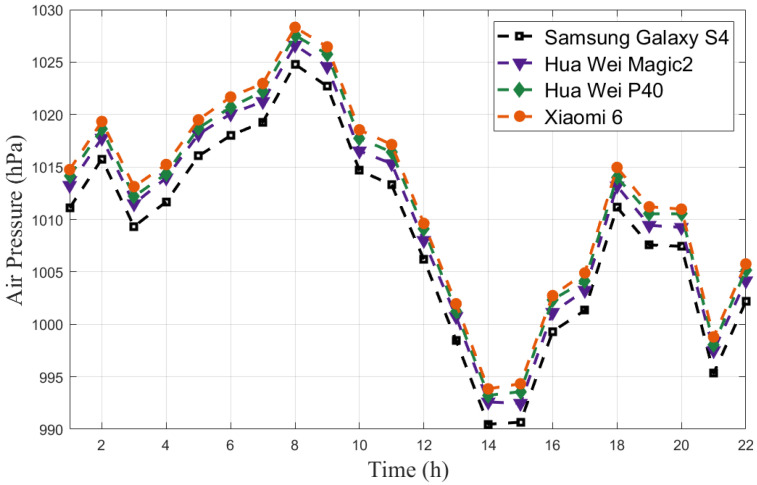
Air pressure variations during a whole day.

Barometric drift is a phenomenon where air pressure changes significantly over time. We collected the air pressure with four smartphones (Samsung Galaxy S4/Hua Wei Magic 2/Hua Wei P40/Xiaomi 6) in a day, as shown in Figure 1. Obviously, air pressure fluctuated greatly over the course of a day, resulting in a difference of up to 4.08 m in altitude. This can cause significant errors in barometer-based altitude measurements.

**Figure 2:**
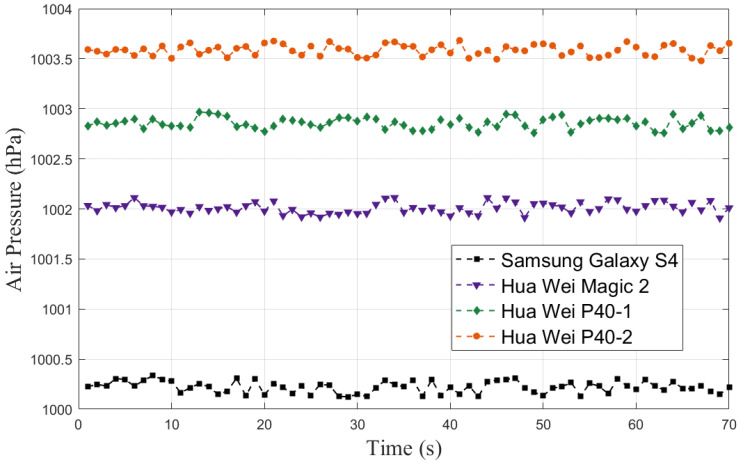
Air pressure variations between different smartphones.

Additionally, equipment heterogeneity can also cause large errors in altimetry. To illustrate equipment heterogeneity more clearly, we collected the air pressure with 4 smart phones (Samsung Galaxy S4/Hua Wei Magic 2/Hua Wei P40-1/Hua Wei P40-2) at the same time, shown in Figure 2. There is a constant difference between the air pressure measurements of different smartphones, even among phones of the same brand. The difference between different brands is about 2–3 hPa, and between the same brand of phones is about 0.5 hPa.

Barometer-based altimetry methods are mostly according to Equations (1) or (2) [5,19], where *p* is the collected air pressure, $p_{0}$ is the reference air pressure, and *t* is the temperature. These methods rely on a fixed reference air pressure, which can maintain high accuracy for a short period of time since the change of air pressure is small in a short time (shown in Figure 2). However, the accuracy will decrease significantly over time due to barometric drift (as shown in Figure 1). Furthermore, equipment heterogeneity limits the widespread use of these methods since a fixed reference air pressure is not suitable for different smartphones.

(1) $$H=44330\times (1-{\left(\frac{100p}{p_{0}}\right)}^{\frac{1}{5.255}})$$

(2) $$H=18400\times \left(1+\frac{t}{273.15}\right)\ln\frac{p_{0}}{p}$$

### 2.2. *Z*-Axis Error of ILS in Anchor-Coplanar Environments

#### 2.2.1. Iterative Least Squares

ILS is an algorithm commonly used in positioning systems that involves iteratively estimating a set of parameters by minimizing the sum of the squares of the differences between observed and predicted values. The algorithm starts with initializing the system, obtaining the initial coordinates of the mobile node $X=\left[\begin{array}{c} x\\ y\\ z \end{array}\right]$ and the locations of anchor $X_{anchor}=\left[\begin{array}{ccc} x_{1} & y_{1} & z_{1}\\ \vdots & \vdots & \vdots \\ x_{n} & y_{n} & z_{n} \end{array}\right]$, and the ranging results between them $D=[d_{1},d_{2},\ldots ,d_{n}]$, where *n* is the number of anchors. The count is set to 0 to start the iteration process. In each iteration, the observation equation is constructed, based on the ranging results and the known locations of the anchors, as Equation (3) (Step (5)).

(3) $$\left[\begin{array}{c} d_{1}\\ \vdots \\ d_{n} \end{array}\right]=\left\{\begin{array}{c} \sqrt{{\left(x_{1}-x\right)}^{2}}+\sqrt{{\left(y_{1}-y\right)}^{2}}+\sqrt{{\left(z_{1}-z\right)}^{2}}\\ \vdots \\ \sqrt{{\left(x_{n}-x\right)}^{2}}+\sqrt{{\left(y_{n}-y\right)}^{2}}+\sqrt{{\left(z_{n}-z\right)}^{2}} \end{array}\right.$$

The Taylor expansion is then performed on the observation equation to linearize it as Equation (4).

(4) $$\left[\begin{array}{c} d_{1}\\ \vdots \\ d_{n} \end{array}\right]=\left[\begin{array}{c} \rho_{1}\\ \vdots \\ \rho_{2} \end{array}\right]+\left[\begin{array}{ccc} \frac{x_{1}-x}{\rho_{1}} & \frac{y_{1}-y}{\rho_{1}} & \frac{z_{1}-z}{\rho_{1}}\\ \vdots & \vdots & \vdots \\ \frac{x_{n}-x}{\rho_{n}} & \frac{y_{n}-y}{\rho_{n}} & \frac{z_{n}-z}{\rho_{n}} \end{array}\right]\cdot \left[\begin{array}{c} \Delta x\\ \Delta y\\ \Delta z \end{array}\right]$$

where $\rho_{i}=\sqrt{{\left(x_{i}-x\right)}^{2}+{\left(y_{i}-y\right)}^{2}+{\left(y_{i}-y\right)}^{2}}$, i=1,2,⋯,n. Assuming $V=\left[\begin{array}{c} d_{1}\\ \vdots \\ d_{n} \end{array}\right]$ - $\left[\begin{array}{c} \rho_{1}\\ \vdots \\ \rho_{n} \end{array}\right]$, $A=\left[\begin{array}{ccc} \frac{x_{1}-x}{\rho_{1}} & \frac{y_{1}-y}{\rho_{1}} & \frac{z_{1}-z}{\rho_{1}}\\ \vdots & \vdots & \vdots \\ \frac{x_{n}-x}{\rho_{n}} & \frac{y_{n}-y}{\rho_{n}} & \frac{z_{n}-z}{\rho_{n}} \end{array}\right]$ and $\Delta =\left[\begin{array}{c} \Delta x\\ \Delta y\\ \Delta z \end{array}\right]$, the Equation (4) can be transferred to Equation (5)

(5) V=A·Δ

*V* represents the difference between the true distance and the estimated distance. To ensure the estimate distance is close to the true distance, *V* needs to be minimized, which is achieved by $V^{T}V=min$ using the principle of least squares. In the algorithm, *V* is calculated at each iteration, and then correction factors Δ are calculated in different directions as shown in Equation (6) (as illustrated in (6))

(6) $$\Delta ={\left(A^{T}A\right)}^{-1}A^{T}V$$

The initial coordinates are updated using the correction vector as $X=\left[\begin{array}{c} x\\ y\\ z \end{array}\right]+\Delta$. The algorithm checks whether the norm of the correction vector is less than a predefined threshold ε. If it is, the algorithm breaks out of the loop; otherwise, the count is incremented, and the iteration continues until the maximum number of iterations (*N*) is reached. The ILS pseudocode is as follows (Algorithm 1).

**Algorithm 1** Traditional Iterative Least Squares  *(1) System initialization*  *(2) Obtain the initial coordinate X, the location of anchors $X_{anchor}$ and ranging results D*  *(3) count = 0;*  *(4) While (count < N and ∥Δ∥ > ε)*  *(5) Construct the observation equation D=F(X)*  *(6) Taylor expansion V=AΔ*  *(7) Calculate the $\Delta ={\left(A^{T}A\right)}^{-1}A^{T}V$*  *(8) Update the initial coordinate X=X+Δ*  *(9) count++*  *(10) End while*

#### 2.2.2. The Effect of Indoor Anchor-Coplanar Environments on *Z*-Axis

ILS positioning involves iteratively calculating Δ and updating the positioning results. To analyze the effect of the indoor anchor-coplanar environment, the iteration trend of Δz needs to be researched. According to ILS, the first derivative of $V^{T}V$ in the *z*-axis direction can be expressed as followw:

(7) $$\frac{\partial V^{T}V}{\partial z}=2\left(\left(z-z_{1}\right)\left(1-\frac{d_{1}}{\rho_{1}}\right)+\left(z-z_{2}\right)\left(1-\frac{d_{2}}{\rho_{2}}\right)+\ldots +\left(z-z_{n}\right)\left(1-\frac{d_{n}}{\rho_{n}}\right)\right)$$

In indoor anchor-coplanar environments, the *z*-axis coordinates are similar ($z_{1}=z_{i}=z_{n}$), so the Equation (7) can be simplified to Equation (8).

(8) $$\frac{\partial V^{T}V}{\partial z}=2\sum_{i=1}^{n}\left(z-z_{i}\right)\left(1-\frac{d_{i}}{\sqrt{{\left(x_{i}-x\right)}^{2}+{\left(y_{i}-y\right)}^{2}+{\left(z_{i}-z\right)}^{2}}}\right)$$

**Figure 3:**
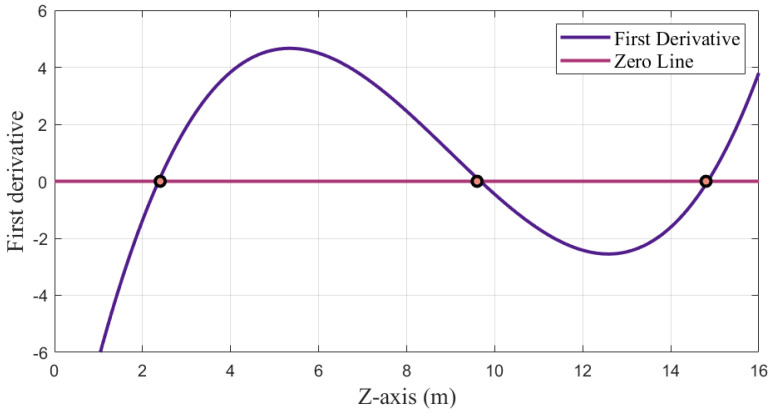
First−order derivative of the objective function in the coplanar direction.

When $z-z_{i}=0$ or ${\left(x_{i}-x\right)}^{2}+{\left(y_{i}-y\right)}^{2}+{\left(z_{i}-z\right)}^{2}=d_{i}$, Equation (8) is equal to 0. Therefore, there are three potential solutions for the *z*-axis coordinate: $z=\tilde{z}$, $z=z_{i}$, or $z=2z_{i}-\tilde{z}$, as illustrated in Figure 3, where $\tilde{z}$ is the true *z*-axis coordinate of the unknown point. This means that in the iteration, the *z*-axis coordinate will update to approach one of the three solutions, and when Δz is less than the threshold, one of the potential solutions will be generated. Assuming that all the anchors are above the tags, the correct solution should be the smallest of the three. However, If the initial iteration point $z_{0}$ is set above the height of anchors, the positioning settlement will fall into the trap of local optimization, and the *z*-axis coordinate may be generated from two solutions other than the correct solution, resulting in a large error (2–3 m). It is worth noting that this analysis is not only applicable to completely coplanar base stations, but also to nearly coplanar ones. When the base station is not completely coplanar, the solution is close to the coplanar solution.

## 3. Proposed Method

### 3.1. System Overview

**Figure 4:**
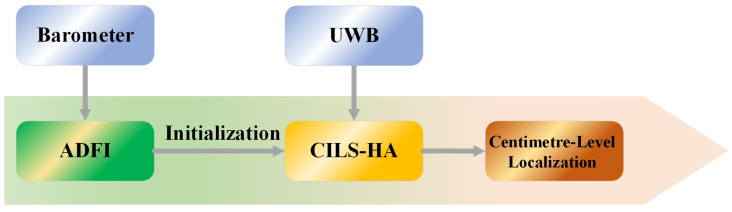
The frame diagram of the proposed method.

The proposed method consists of two main steps, as shown in Figure 4. Firstly, we introduce ADFI to achieve precise floor identification. Then, in the second step, we propose the CILS-HA algorithm, which uses the results of the floor identification algorithm from Step 1 to complete the initialization process and perform indoor altimetry using UWB technology. ADFI combines traditional barometer-based altimetry with activity detection to minimize the impact of equipment heterogeneity and barometric drift. Meanwhile, CILS-HA enhances the *z*-axis accuracy by restricting the range of the initial *z*-axis coordinate and the step length and direction of the iteration.

### 3.2. ADFI

To solve the equipment heterogeneity and barometric drift, we propose the ADFI algorithm. The algorithm utilizes activity detection to distinguish upstairs, downstairs, and horizontal walking. The clear description of activity detection and its experimental basis are introduced as follows.

**Figure 5:**
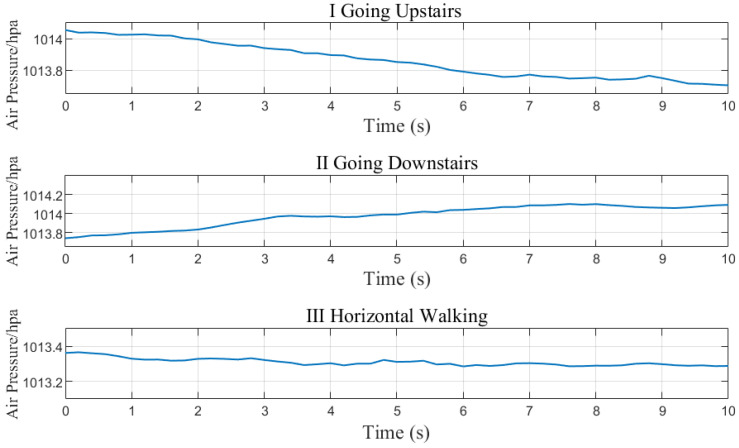
Air pressure changes when going upstairs, downstairs, and walking horizontally.

Figure 5 shows the change in air pressure over time (sampling frequency is 5 Hz) in three behavioural states: going upstairs, downstairs, and horizontal walking. The fluctuation of air pressure caused by horizontal movement is much smaller than that caused by vertical movement as shown in Table 1.

**Figure 6:**
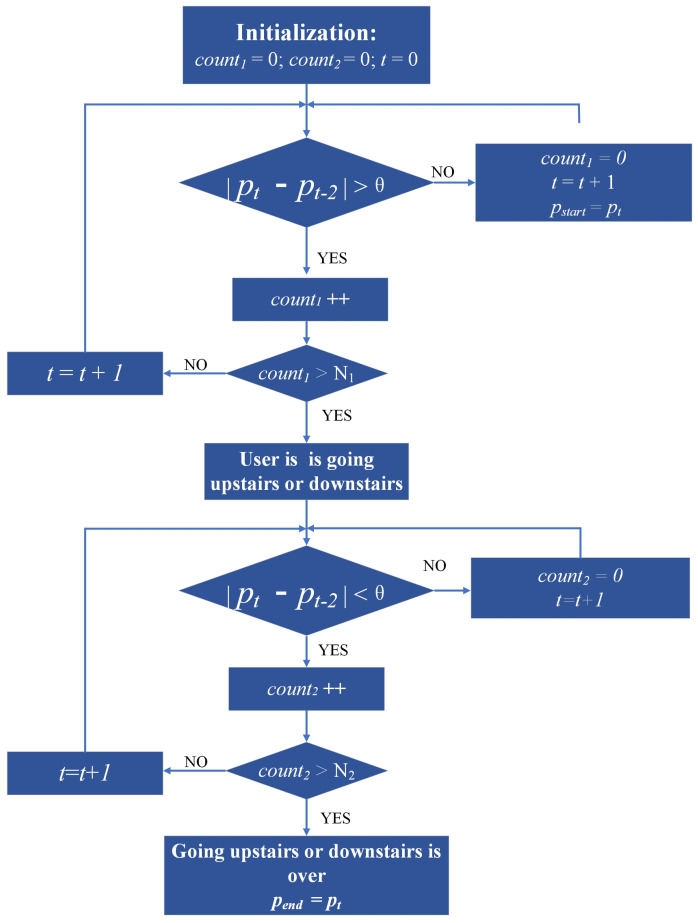
Activity detection flow chart.

According to the above analysis, an activity detection algorithm is proposed, as shown in Figure 6, that utilizes counting variables $count_{1}$ and $count_{2}$, an air pressure difference threshold θ, time stamp *t*, air pressure value $p_{t}$ at time *t*, and air pressure values $p_{start}$ and $p_{end}$, which are recorded at the beginning and end of the going upstairs or downstairs. When the air pressure difference between the last two seconds exceeds the threshold θ, $count_{1}$ is incremented; otherwise, it is reset. When $count_{1}$ exceeds $N_{1}$, going upstairs or downstairs is identified. Similarly, when the air pressure difference between the last two seconds falls below the threshold θ, $count_{2}$ is incremented; otherwise, it is reset. When $count_{2}$ exceeds $N_{2}$, the end of going upstairs or downstairs is identified. Then, the $p_{start}$ and $p_{end}$ are used to calculate the height, according to Equation (11), where $p_{start}$ is the reference air pressure $p_{0}$ and $p_{end}$ is the collected air pressure *p*. Finally, based on the estimated height and the height differences between floors, the floor number can be determined.

Real-time updating of the reference air pressure in our proposed algorithm eliminates the effect of barometric drift. Additionally, as shown in Figure 1 and Figure 2, there are fixed differences between the air pressure collected by different smartphones, but the changes in air pressure are almost identical. This means that the estimated heights of different smartphones based on our proposed method are nearly the same, demonstrating that equipment heterogeneity has been solved. However, it should be noted that the accuracy of the estimated height using our ADFI is low due to the low accuracy of the barometers embedded in smartphones. Therefore, the estimated height can only be used for floor identification and not for accurate localization.

### 3.3. CILS-HA

According to the analysis above, the selection of initial *z*-axis coordinate is crucial for the *z*-axis localization. When the anchors are deployed at similar heights, the potential *z*-axis coordinates from ILS are $\tilde{z}$, $z_{i}$, or $2z_{i}-\tilde{z}$, sorted from smallest to largest. To ensure convergence to the correct value, the initial *z*-axis coordinate should be set below the true height of the user ($\tilde{z}$), whereas coordinates between $\tilde{z}$ and $z_{i}$ may result in values between those heights. If the initial coordinate is higher than the anchor height, the potential coordinates can fall between $z_{i}$ and $2z_{i}-\tilde{z}$. Therefore, we propose setting the initial *z*-axis coordinate as the height of the current floor ($z_{0}=h_{floor}$), provided by ADFI. When all anchors are below the tag, the highest potential coordinate is correct, and the initial coordinate should be set above $z_{i}$. However, when the anchors are on a floor below the user, the ranging accuracy of UWB is poor due to non-line-of-sight (NLOS), so this paper selects only the anchors on the current floor to improve accuracy.

In order to further ensure that the *z*-axis coordinates can converge to the true coordinate $\tilde{z}$, the iteration direction and step size are constrained. As analyzed above, with the initial coordinate of the *z*-axis set to the height of the current floor, the *z*-axis coordinate will increase monotonically during convergence. However, only constraining the iteration direction may lead to incorrect convergence. According to Equation (9), the iteration step of ILS is 1, and the size of the iterative update value is determined by the first- and second-order derivative of the function. When the coordinate convergence is close to the correct value, the value of the first derivative gradually decreases, and the reciprocal of the second derivative increases, resulting in a larger Δ. After updating, the estimated coordinate value may be greater than the correct value and cannot converge to the correct value due to the constrain of iteration direction. To avoid this, the step size is also constrained, with a step coefficient set as Equation (10) and *C* a nonnegative constant, considering the centimeter-level positioning accuracy.

(9) $$X_{k+1}=X_{k}+{\left(\frac{\partial^{2}V^{T}V}{\partial X^{2}}\right)}^{-1}\left(\frac{\partial V^{T}V}{\partial X}\right)$$

(10) $$W=\left[\begin{array}{ccc} 10^{-c} & 0 & 0\\ 0 & 10^{-c} & 0\\ 0 & 0 & 10^{-c} \end{array}\right]$$

After the adjustment, all three directional components are updated to the centimeter level, which can be expressed as:

(11) $$\left[\begin{array}{c} x_{k+1}\\ y_{k+1}\\ z_{k+1} \end{array}\right]=\left[\begin{array}{c} x_{k}\\ y_{k}\\ z_{k} \end{array}\right]+W\left[\begin{array}{ccc} \frac{\partial^{2}V^{T}V}{\partial x^{2}} & \frac{\partial^{2}V^{T}V}{\partial xy} & \frac{\partial^{2}V^{T}V}{\partial xz}\\ \frac{\partial^{2}V^{T}V}{\partial yx^{2}} & \frac{\partial^{2}V^{T}V}{\partial y^{2}} & \frac{\partial^{2}V^{T}V}{\partial yz}\\ \frac{\partial^{2}V^{T}V}{\partial zx} & \frac{\partial^{2}V^{T}V}{\partial zy} & \frac{\partial^{2}V^{T}V}{\partial z^{2}} \end{array}\right]\left[\begin{array}{c} \frac{\partial V^{T}V}{\partial x}\\ \frac{\partial V^{T}V}{\partial y}\\ \frac{\partial V^{T}V}{\partial z} \end{array}\right]$$

By selecting the initial coordinate and applying the iteration constraints, the *z*-axis coordinate can converge to the correct value $\tilde{z}$. The pseudocode of CILS-HA is shown as follows. In the system initialization (Step (1)), the initial *z*-axis coordinate is set as the height of the floor ($h_{f}loor$). Once achieving Δ, the convergence direction and step size are constrained (Step (8) to Step (12)).

The proposed method in this paper has two categories for setting the iteration termination condition: the first is when the difference between the objective function (Δ) of two consecutive iterations is less than a threshold (ε), and the second is when the number of iterations exceeds the maximum iteration number *N*. In the algorithm proposed, the objective function has extreme values, and the iteration can converge by primarily relying on the first type of iteration termination condition, that is, Δ<ε. While *N* is set to 30, actual tests revealed an average of 15 iterations and a maximum of 18 iterations for 100 sets of data. As such, the maximum iteration value *N* is only for risk avoidance and is typically not triggered. During the iterative calculation process, Δ monotonically decreases, leading to *X* gradually converging to the final result after Δ<ε. Determining ε involves considering four factors: positioning accuracy, number of iterations, system error and noise, and dataset size and complexity. Since the positioning accuracy requirement is at the centimeter level, ε must be set to a small value. However, if ε is too small, the algorithm may iterate too many times, leading to an increased running time. System error and noise are relatively small due to the antimultipath capability of UWB, while the dataset’s size and complexity are low. Multiple tests showed that setting ε to $e^{-7}$ leads to an average of 15 iterations and less than 1 s of average positioning time, meeting the centimeter-level accuracy requirement. The pseudocode of the proposed CILS algorithm is shown below (Algorithm 2).

**Algorithm 2** Constrained Iterative Least Squares  *(1) System initialization: Initial coordinate X=(x,y,z), $z=h_{floow}$; Step length W*  *(2) Obtain the location of anchors $X_{anchor}$ and ranging results D*  *(3) count = 0;*  *(4) While (count < N and ∥Δ∥  <  ε and ∥Δ∥  >  ε)*  *(5) Construct the observation equation D=F(X)*  *(6) Taylor expansion V=AΔ*  *(7) Calculate the $\Delta ={\left(A^{T}A\right)}^{-1}A^{T}V$*  *(8) Update the initial coordinate X=X+WΔ*  *(9) count++*  *(10) End while*

## 4. Experiments and Analysis

### 4.1. Environmental Setup and Data Collection

#### 4.1.1. Environmental Setup

**Figure 7:**
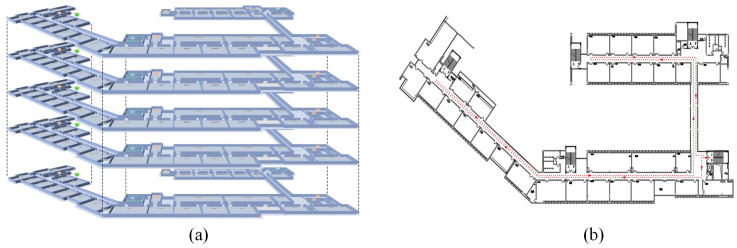
The deployment of teaching building (**a**) the structure of the teaching building. (**b**) plan of each floor.

ADFI and CILS-HA were evaluated through two experiments in a teaching building and a laboratory building. Figure 7a shows the structure of the teaching building, with a plan of each floor shown in Figure 7b. The building has five floors, with heights between each floor of 5 m, 4 m, 4 m, and 5 m, respectively. The building is equipped with 44 UWBs, 56 Wi-Fis, and 43 BLEs, allowing for comparison of the proposed ADFI and CILS-HA with existing Wi-Fi and BLE-based methods. The numbers of UWBs, Wi-Fis, and BLEs on each floor are similar.

**Figure 8:**
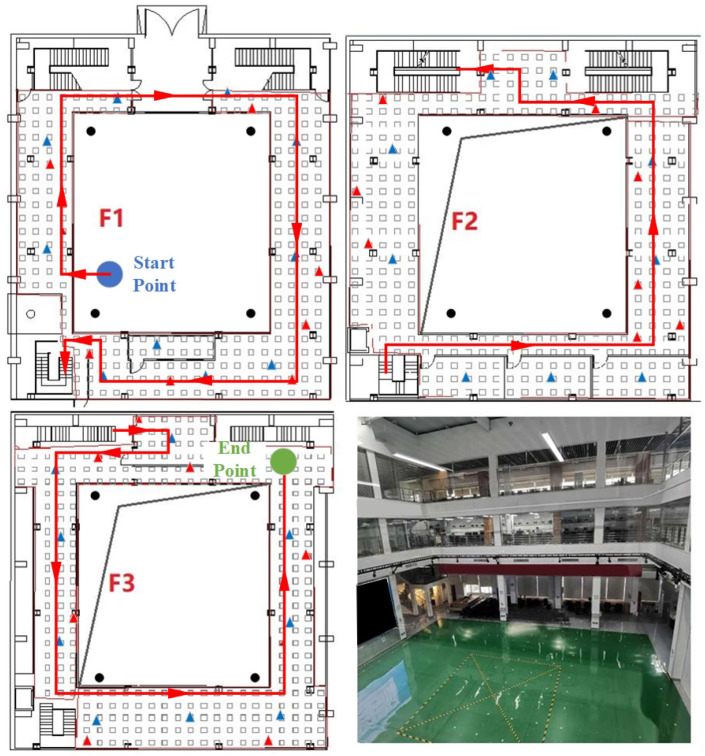
The deployment of laboratory building.

Figure 8 shows the laboratory building, where Wi-Fi is represented by blue triangles, BLE by red triangles, and UWB by black circles. Each floor is equipped with 8 Wi-Fis and 10 BLEs, and 4 UWBs are installed on the ceiling of the building. The heights between each floor are 5.5 m and 2.5 m. Compared to the teaching building, the laboratory building is more challenging than the teaching building for two reasons: (1) the height difference between the second and third floors is small, resulting in a smaller air pressure difference compared to the teaching building; and (2) the middle part of each floor is a hollow area (as shown in Figure 8), which leads to less obvious signal differences between floors compared to the teaching building.

#### 4.1.2. Data Collection for ADFI

For the ADFI experiment, 325 Wi-Fi reference points were set in the teaching building and 144 Wi-Fi reference points were set in the laboratory to enable comparison with other Wi-Fi-based methods. During data collection, the tester walked with a mobile phone along the red line shown in Figure 7 and Figure 8 and collected real-time air pressure and Wi-Fi data.

#### 4.1.3. Data Collection for CILS-HA

In the CILS-HA experiment, the altimetry accuracy of the algorithm was tested using a fixed smartphone placed on a tripod at a height of 1.3 m. In addition to the Wi-Fi reference points, BLE reference points were also set up with the same number and positions as the Wi-Fi reference points. The experiment also involved setting up 48 testing points in the teaching building and 100 testing points in the laboratory. During data collection, the tester collected air pressure, UWB, Wi-Fi, and BLE data at the test points on each floor, starting from the first floor. Each testing point was sampled for 15 s at a rate of 20 Hz.

### 4.2. Performance of ADFI

To verify the performance of ADFI, we conducted two experimental campaigns and compared them with three existing algorithms in literature: Bayesian classification-based floor Identification (BCFI) [5], Viterbi [8], and the adaptive unscented Kalman filter (AUKF) [20]. BCFI and Viterbi are based on Wi-Fi, and AUKF is based on barometers.

#### 4.2.1. Dynamic Experiment

**Figure 9:**
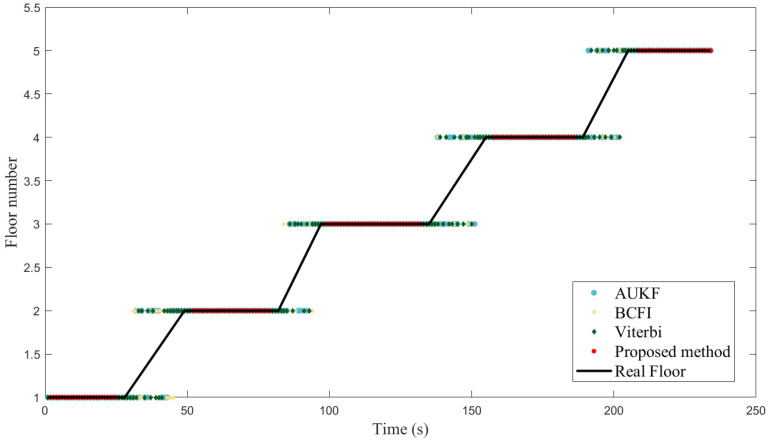
Experiment results in teaching building.

**Figure 10:**
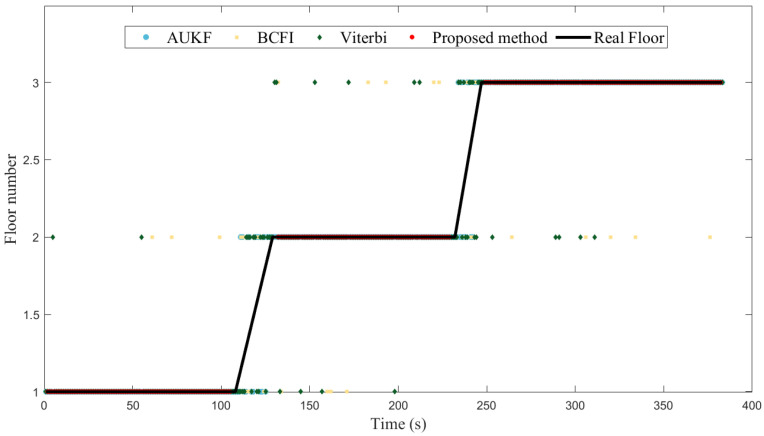
Experiment results in laboratory building.

Figure 9 and Figure 10, respectively, show the dynamic experimental results of the proposed method and the comparative methods in two different environments. In a short period of time, the barometric pressure remains relatively constant, so both the AUKF method and the proposed method can maintain a high accuracy of 99.54% and 99.33%, respectively. Compared to AUKF, the proposed method incorporates the activity detection based on the barometric sequence, resulting in a delay of approximately 1.5 s, and thus the accuracy is slightly lower. However, compared with the AUKF method, our method does not show repeated transformations in floor identification results when walking up and downstairs, as our method incorporates activity detection. The Wi-Fi signal-based methods, including BCFI and Viterbi, have an accuracy of 100% when walking on the plane in the teaching building, but drop to 87.33% and 83.64%, respectively, in the laboratory building. Especially on the second and third floors, due to the close proximity of the floors and the existence of hollow areas, the signal differences between floors are not obvious, and the Wi-Fi-based floor identification methods sometimes fail to distinguish signal features. Meanwhile, BCFI and Viterbi, like the AUKF, also show repeated transformations in floor identification results when walking up and down stairs, while our method does not, demonstrating its innovation.

#### 4.2.2. Static Experiment

**Figure 11:**
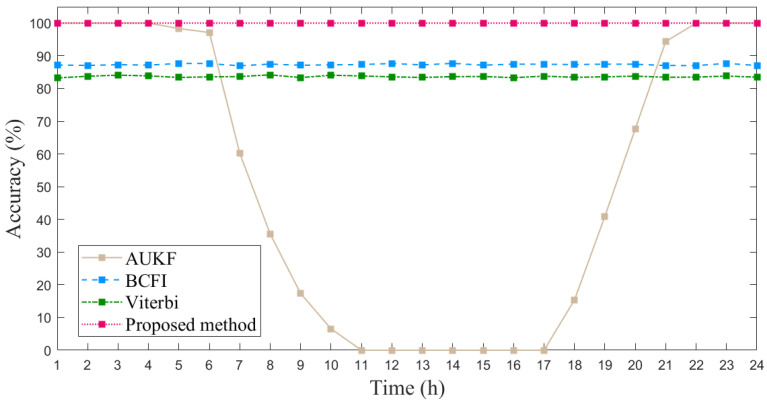
The identification accuracy of four methods in a whole day.

We also conducted a static experiment to test the stability of the method and its ability to eliminate the influence of barometric drift. We placed a phone on the plane of the second floor and left it stationary for a whole day, collecting Wi-Fi, Bluetooth, and barometer data and outputting the floor identification results. Figure 11 illustrates the average accuracy of floor identification achieved by four methods over different time intervals. Our method achieved a 100% identification accuracy, as it incorporates an activity detection module that does not perform floor identification when no activity is detected, resulting in consistent and unchanged output with 100% accuracy. This indicates that when the phone is stationary, the floor identification results will not be affected by barometric drift and remain unchanged. However, it should be noted that this does not guarantee the real-time identification accuracy of our method to maintain 100%. The accuracy of the AUKF method based on the barometer can still maintain 100% accuracy within 4–5 h, but with time passing, the barometer data will change significantly, causing its accuracy to drop to 0 in a short period. The reason why the accuracy of our proposed method and the AUKF method can maintain almost 100% within 4 h is that the barometric pressure at the same location remains relatively constant over a short period of time when they remain stationary. The accuracy of the other two methods, BCFI and Viterbi, also remains basically unchanged, as they do not rely on barometer data and are not affected by pressure drift.

### 4.3. Performance of CILS-HA

We conducted a positioning experiment in both the teaching building and laboratory building to verify our theoretical analysis of *Z*-axis error in anchor-coplanar environments. We compared the traditional ILS-based positioning accuracy of the three axes (x, y, z), and the results are shown in Table 2. The accuracy of the *z*-axis was significantly worse than that of the *x*-axis and *y*-axis when the anchors were installed with a similar height difference in an indoor environment, demonstrating the correctness of our analysis. The accuracy difference between the two scenarios was due to the UWB signals from anchors to tags being blocked by glasses and concrete walls in the laboratory building, resulting in a serious NLOS effect.

**Figure 12:**
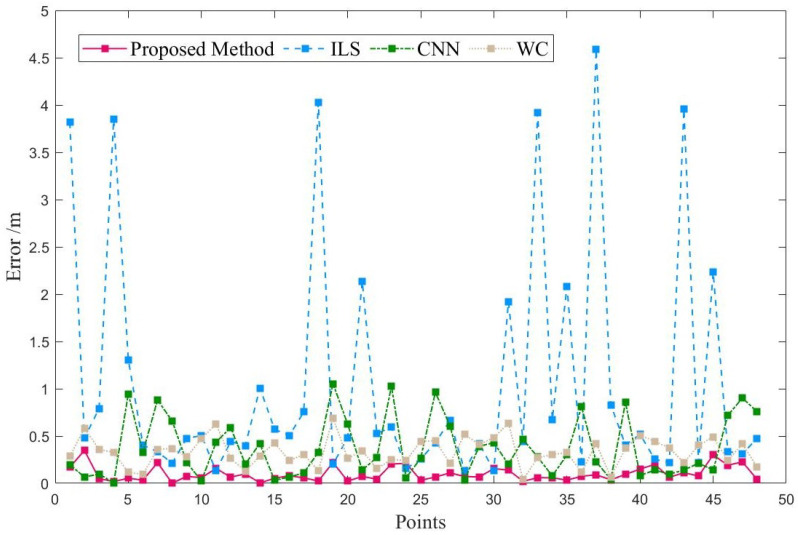
Positioning error of each point in teaching building.

To evaluate the performance of our proposed method, we compared it with two existing methods: 1-D CNN (based on BLE) [21] and WC (based on Wi-Fi fine-time-measurement) [22], as well as the traditional ILS. Figure 12 shows the positioning error at each testing point in the teaching building. Although the traditional ILS achieves similar positioning accuracy at some testing points, there are still ten errors of more than 1 m, which is due to the anchor-coplanar environment. In contrast, the proposed method achieves positioning errors of less than 0.5 m at all testing points. Furthermore, compared with CNN and WC, the proposed method achieves lower errors at most points.

**Figure 13:**
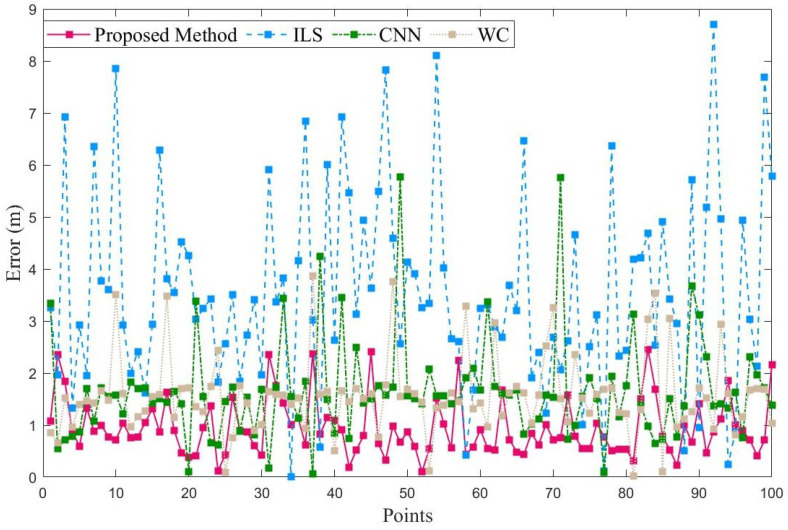
Positioning error of each point in laboratory.

Figure 13 shows the positioning error at each testing point in the laboratory. However, as in the teaching building, our proposed method maintains the fewest errors among these methods. Notably, compared to CNN and WC, the number of errors greater than 2 m is smaller, demonstrating the robustness of our proposed method. Compared to the teaching building environment, the laboratory environment is more confined, but there are more wireless signal devices such as Wi-Fi, Bluetooth, and UWB deployed on each floor, and there is also more metal equipment, resulting in more interference and severe multipath effects. Therefore, the UWB ranging error in the laboratory environment is larger than that in the teaching building environment.

Table 3 presents the *Z*-axis positioning errors of the four methods in the teaching building. The proposed method yielded a mean error (ME) of 0.101 m, which is better than ILS, CNN, and WC by 0.84 m, 0.288 m, and 0.248 m, respectively. Moreover, the proposed method produced a root-mean-square error (RMSE) that was 1.162 m, 0.266 m, and 0.252 m smaller than ILS, CNN, and WC, respectively. Meanwhile, Table 4 shows the *Z*-axis positioning errors of the four methods in the laboratory. The proposed method attained an ME of 0.947 m, which is better than ILS, CNN, and WC by 2.607 m, 0.696 m, and 0.625 m, respectively. The proposed method also achieved an RMSE that was 3.491 m, 0.496 m, and 0.361 m smaller than ILS, CNN, and WC, respectively. These results indicate that the proposed method surpassed the other three methods in terms of accuracy. Remarkably, in the NLOS environment, ILS, CNN, and WC had an increase of 2.603 m, 1.254 m, and 1.223 m, respectively, in their ME, while the proposed method only increased by 0.846 m. Likewise, the RMSE of ILS, CNN, and WC increased by 3.477 m, 1.378 m, and 1.284 m, respectively, whereas the proposed method’s RMSE increased by only 1.148 m. These findings support the conclusion that the proposed method is more robust than the other three methods in NLOS environments.

**Figure 14:**
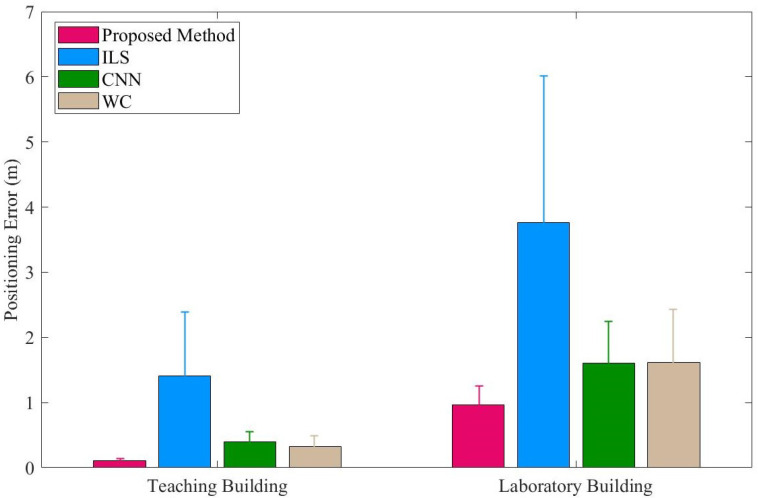
Positioning error of four methods in teaching building and laboratory.

Figure 14 displays the error bars for the proposed method, ILS, CNN, and WC in both the teaching building and laboratory. The error bar for the proposed method is shorter than those of the other three methods, indicating that the standard deviation (STD) of the proposed method is smaller. This suggests that the performance of the proposed method is more stable than that of the other methods.

**Figure 15:**
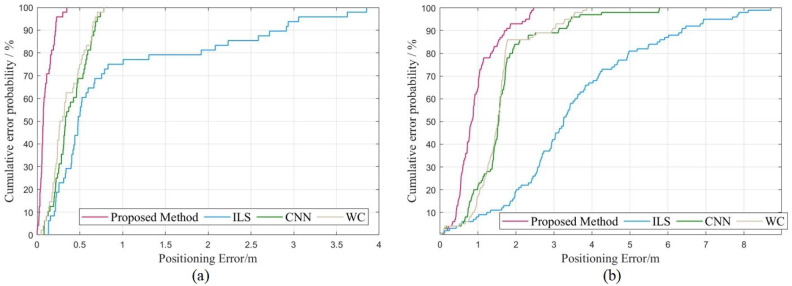
CDF of four methods (**a**) in teaching building. (**b**) in laboratory.

Figure 15a,b depict the cumulative probability of the proposed method, ILS, CNN, and WC. In the teaching building, 80% of the proposed method’s errors are less than 0.169, whereas the corresponding values for ILS, CNN, and WC are less than 1.918, 0.625, and 0.549, respectively. In the laboratory, 80% of the proposed method’s errors are less than 1.369, while the corresponding values for ILS, CNN, and WC are less than 4.967, 1.907, and 1.714, respectively. These results demonstrate that the *Z*-axis positioning errors of the proposed method are smaller than those of ILS, CNN, and WC.

**Figure 16:**
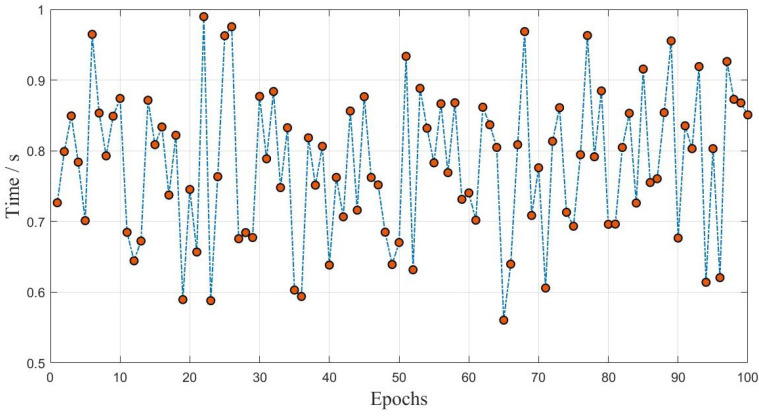
Time consumption of proposed method.

Finally, assessing the time efficiency of the proposed method is crucial. Compared to the traditional ILS, our method requires more iterations due to the step length constraint. The proposed method takes an average of 15 iterations, whereas ILS takes about 6. Figure 16 illustrates the time consumption of the proposed method. The majority of epochs take between 0.7 s and 0.9 s, and 11 epochs take more than 0.9 s, but all are completed in less than 1s, satisfying the real-time positioning requirement.

## 5. Discussion and Future Work

Indoor altimetry plays a critical role in commercial and industrial buildings. However, despite the maturity of indoor 2D positioning, accurate smartphone-based indoor 3D altimetry methods are still lacking. To address this, our paper proposes a hybrid barometer and UWB technology-based altimetry method. We optimize the floor identification method using barometers and the iterative ILS method. To evaluate the performance of our two methods, tests were conducted in a teaching building and a laboratory. The experimental results show that our activity detection-based floor identification method outperforms other methods, effectively eliminating repeated transformations during the up- and downstairs process in dynamic experiments and the effect of barometric drift in static experiments. Additionally, our proposed altimetry method based on constrained ILS achieves improved positioning accuracy of 0.84 m, 0.288 m, and 0.248 m in the teaching building compared to ILS, 1D-CNN, and WC, respectively, and 2.607 m, 0.696 m, and 0.625 m in the laboratory.

For future work, we plan to further integrate the proposed indoor altimetry with plane positioning to form a complete 3D positioning system. Currently, many scholars have carried out a lot of work in UWB-based 2D positioning [23], using various machine learning algorithms to reduce the impact of non-line-of-sight and clock drift on UWB positioning [24,25]. Precise indoor height measurement technology can provide sufficient prior information for 2D positioning, such as excluding some UWB base stations with poor signal quality from other floors. Through this tightly coupled approach, it may be possible to further improve the positioning accuracy of UWB and achieve ubiquitous indoor positioning.

## Figures and Tables

**Table 1:** The air pressure difference within 2 s under three different activities.

Average Air Pressure Differences	Activity		
	Going Upstairs (hpa)	Going Downstairs (hpa)	Horizontal Walking (hpa)
$|p_{0}-p_{2}|$	0.06	0.05	0.01
$|p_{2}-p_{4}|$	0.07	0.06	0.02
$|p_{4}-p_{6}|$	0.08	0.07	0.01
$|p_{6}-p_{8}|$	0.07	0.08	0.01
$|p_{8}-p_{10}|$	0.09	0.10	0.01

**Table 2:** Comparison of positioning accuracy in the x, y, and z axes in the teaching building and laboratory.

Axes	X (m)	Y (m)	Z (m)
Teaching building	0.181	0.227	1.404
Laboratory building	0.932	1.077	3.743

**Table 3:** Accuracy of four methods in teaching building.

Algorithm	50%	70%	80%	ME (m)	RMSE (m)
Proposed Method	0.071	0.112	0.169	0.101	0.144
ILS	0.481	0.754	1.918	0.941	1.306
CNN	0.332	0.536	0.625	0.389	0.410
WC	0.266	0.472	0.549	0.349	0.396

**Table 4:** Accuracy of four methods in laboratory.

Algorithm	50%	70%	80%	ME (m)	RMSE (m)
Proposed Method	0.820	1.022	1.369	0.947	1.292
ILS	3.250	4.184	4.967	3.554	4.783
CNN	1.519	1.710	1.907	1.643	1.788
WC	1.546	1.653	1.714	1.572	1.653

## Data Availability

The data used in this paper were obtained through measurements by the authors. No publicly available data are cited, and data sharing is not applicable to this article.
